# Starch-dependent sodium accumulation in the leaves of *Vigna riukiuensis*

**DOI:** 10.1007/s10265-023-01470-8

**Published:** 2023-05-26

**Authors:** Yusaku Noda, Atsushi Hirose, Mayumi Wakazaki, Mayuko Sato, Kiminori Toyooka, Naoki Kawachi, Jun Furukawa, Keitaro Tanoi, Ken Naito

**Affiliations:** 1 National Institutes for Quantum Science and Technology (QST), Takasaki Institute of Advanced Quantum Science, Gunma, 370-1292 Japan; 2 Research Center of Genetic Resources, National Agriculture and Food Research Organization, Ibaraki, 305-8602 Japan; 3 Hoshi University, Tokyo, 142-8501 Japan; 4 Mass Spectrometry and Microscopy Unit, RIKEN Center for Sustainable Resource Science, 230-0045 Kanagawa, Japan; 5 Faculty of Life and Environmental Sciences, University of Tsukuba, Ibaraki, 305-8572 Japan; 6 Graduate School of Agricultural and Life Sciences, The University of Tokyo, Tokyo, 113-8657 Japan

**Keywords:** Autoradiography, Genus Vigna, Salt tolerance, Starch granules, Wild crop relatives

## Abstract

This research provides insight into a unique salt tolerance mechanism of *Vigna riukiuensis*. *V. riukiuensis* is one of the salt-tolerant species identified from the genus *Vigna*. We have previously reported that *V. riukiuensis* accumulates a higher amount of sodium in the leaves, whereas *V. nakashimae*, a close relative of *V. riukiuensis*, suppresses sodium allocation to the leaves. We first suspected that *V. riukiuensis* would have developed vacuoles for sodium sequestration, but there were no differences compared to a salt-sensitive species *V. angularis*. However, many starch granules were observed in the chloroplasts of *V. riukiuensis*. In addition, forced degradation of leaf starch by shading treatment resulted in no radio-Na (^22^Na) accumulation in the leaves. We performed SEM–EDX to locate Na in leaf sections and detected Na in chloroplasts of *V. riukiuensis*, especially around the starch granules but not in the middle of. Our results could provide the second evidence of the Na-trapping system by starch granules, following the case of common reed that accumulates starch granule at the shoot base for binding Na.

## Introduction

Salt tolerance has been one of the most important issues in plant science as soil salinity is a major constraint of crop production. In addition, given a rapid depletion of ground water plus the rapid growth of global population, there is a concern that demands for drinking water may compete with those for food production (Wada et al. 2010). Thus, there is a growing demand for salt-tolerant crops, which can be grown with salinized water (Panta et al. 2014).

To this goal, we have screened genetic resources of wild Vigna species and identified several species that are highly tolerant to salt stress (Iseki et al. 2016; Yoshida et al. 2016). The genus *Vigna* is a reservoir of diversity and many species are adapted to harsh environments, such as marine beach, desert, limestone karsts and marshes (Tomooka et al. 2014; van Zonneveld et al. 2020). As expected, the species collected from coastal areas presented high tolerance to salt (Iseki et al. 2016).

The following studies on sodium allocation have revealed that *Vigna riukiuensis* (Ohwi) Ohwi & H.Ohashi accumulates a relatively higher amount of sodium in the leaves, whereas *Vigna nakashimae* (Ohwi) Ohwi & H.Ohashi*,* a close relative of *V. riukiuensis*, suppresses sodium accumulation to the leaves (Noda et al. 2022; Yoshida et al. 2016). Thus, we have hypothesized that *V. riukiuensis* has a mechanism of salt-includer, which isolates sodium into vacuoles to lower sodium concentration in cytoplasm.

To confirm this hypothesis, in this study, we first observed vacuoles of the leaf cells to see whether there are any structural differences between *V. riukiuensis* and other species. However, we could not find any characteristic features in the vacuoles. Instead, we found an abundance of starch granules in the chloroplast of *V. riukiuensis* leaves. Since there has been a report that common reed accumulates starch granules that bind Na at the shoot base (Kanai et al. 2007), we were intrigued to test whether the starch granules in *V. riukiuensis* also have ability to bind Na. To do so, we performed further experiments including element mapping.

## Materials and methods

### Plant material and growth conditions

All plant seeds were provided by the NARO Genebank in Tsukuba, Japan (https://www.gene.affrc.go.jp/index_en.php). Seeds were germinated on Seramis clay (Westland Deutschland GmbH, Mogendorf, Germany) for 1 week and then transferred to hydroponic solution in a growth chamber (Light: 28 °C for 14 h and Dark: 25 °C for 10 h. Light intensity 500 µM^−1^ s^−1^ m^−2^) until autoradiographic imaging. The hydroponic solution contained a diluted nutrient solution of a 1:1 ratio of OAT House No.1 (1.5 g L^−1^): OAT House No.2 (1 g L^−1^) (Otsuka Chemical Co, Japan), which contained 18.6 mEq L^−1^ N, 5.1 mEq L^−1^ P, 8.6 mEq L^−1^ K, 8.2 mEq L^−1^ Ca and 3.0 mEq L^−1^ Mg.

### Visualization of ^22^Na

Pre-cultured plants were transplanted to a new hydroponic solution containing 5 kBq ^22^Na (PerkinElmer, USA) with non-radioactive 100 mM ^23^NaCl. After adding the radio-isotope, plants were incubated again in a long-day condition (Light: 28 °C for 14 h and Dark: 25 °C for 10 h. Light intensity 200 µmol s^−1^ m^−2^) for 3 days. After incubation, we carefully washed the roots and then enclosed the whole plant body into a plastic bag, and exposed it to a Storage Phosper Screen (BAS-IP-MS-2025E, GE Healthcare, UK) in Amersham exposure cassettes (GE Healthcare, UK) for 24 h. We then scanned the exposed screen with a laser imaging scanner Typhoon FLA-9500 (GE Healthcare, UK). To arrange radioactive intensity equally at each image, photo-stimulated luminescence and contrast were equalized by Multi Gauge version 3.0 (Fujifilm, Japan). All the experiments were independently done on more than three biological replicates.

### ICP-MS

We germinated the seeds on Seramis clay, cultivated for 1 week and then transferred 4 plants of each species to hydroponic solution (as described above) in a growth chamber (Light: 28 °C for 14 h and Dark: 24 °C for10 h). When the 3rd leaves had fully expanded, we transferred the plants to hydroponic culture with 100 mM NaCl for 2 days. After incubation, we separately collected the 1st and 2nd leaves and dried at 50 °C for 3 days. The leaves were digested with 200 µL 69% HNO_3_ at 90 °C for 0.5 h. The digestate was diluted to 1-in-140 with Milli-Q water and inductively coupled plasma-mass spectrometry (ICP-MS, NexION 350S, PerkinElmer, Waltham, MA, USA) determined the contents of Na. The Tukey HSD test was used to compare differences in the measured variables of leaf Na and K concentration, respectively. Differences were significant when *p* < 0.05.

### Starch staining and correlation between starch and Na accumulation with shading in *V. riukiuensis*

#### Iodine staining

At the beginning of the treatment, we grew *Vigna angularis* (Willd.) Ohwi & H.Ohashi*,, V. nakashimae* and *V. riukiuensis* in hydroponic solution for 7 days, 14 days and 21 days, respectively, to unify the growth stage of the plants. Plants were grown in 100 mM NaCl hydroponic solution or non-NaCl solution for 3 days. After incubation, we harvested fresh leaves, immediately immersed them in hot water for 10 min and decolorized in 99.5% ethanol for 5 min. Finally, samples were immersed 1/50 iodine solution (20 g L^−1^ KI, 10 g Iodine) for 10 min.

#### Shading experiment

*V. riukiuensis* plants were grown in hydroponic solution for 21 days. The middle leaflets of the 1^st^, 2^nd^ and 3^rd^ leaves were masked with aluminum foil (shaded) or cling film (non-shaded control) at 2 pm. Plants were transferred to 100 mM ^22^NaCl solution (5 kBq) for 72 h under long-day condition. After ^22^Na treatment, ^22^Na localization was visualized using the method described above. For testing the effect of salt stress on starch degradation, we transferred the 3-week-old plants to 100 mM ^23^NaCl solution for 24 h and then covered the leaflets with aluminum foil or cling film at 2 pm and kept the plants in 100 mM NaCl for another 24 h. Leaves were then collected and processed for iodine–starch staining as described above.

### Electron microscopy and SEM–EDX

We treated *V. angularis*, *V. nakashimae* and *V. riukiuensis* with 100 mM NaCl for 3 days. We cut the leaves into 5 × 5 mm^2^ pieces, and the samples were fixed with 4% (w/v) paraformaldehyde and 2% (v/v) glutaraldehyde in 50 mM cacodylate buffer for 2.5 h at room temperature. The samples were washed six times by the same buffer and postfixed with 1% osmium tetroxide in 50 mM cacodylate buffer for 2 h. After washing with double distilled water, the samples were dehydrated in a methanol series (25, 50, 75, 90, 100%) and substituted to methanol:propylene oxide (1:1) to 100% propylene oxide. Next, the samples were substituted in propylene oxide:　Epon812 series (3:1, 1:1, 1:3) and finally embedded in 100% Epon812 resin (TAAB, UK). Semi-thin sections (1 or 2 µm) were cut with a diamond knife on an ultramicrotome (EM UC7, Leica Microsystems, Germany). To test whether there are any structural differences between each species, 1 µm thick sections were observed by a field-emission scanning electron microscope (FE-SEM). Sections were dried on glass slides and stained with 0.4% uranyl acetate and lead citrate solution. Sections were coated with osmium tetroxide with an osmium coater (HPC-1SW, Vacuum device, Japan), then observed by FE-SEM (SU8220, Hitachi High-Tech, Japan) at accelerating voltage 5 kV with an yttrium aluminum garnet backscattered electron detector. For scanning electron microscope-energy dispersive x-ray spectrometry (SEM–EDX), 2 µm thickness sections were dried on carbon tape at 60 °C and set on the scanning electron microscope (SEM) stub. The Na imaging condition was high vacuum and acceleration voltage mode as follows: Acceleration voltage, 15 kV; Spot intensity, 80; Beam exposure time, 1 h. As beam irradiation causes a slight elongation of the sections, electron microscopy images were acquired after beam irradiation in low vacuum and acceleration voltages. Spectral acquisition of Na and electron microscope images were acquired by EMAX Evolution (version EMAX 2.2 SP2, HORIBA, Japan) and SU3500 (Hitachi High-Tech, Japan), respectively. Spectral data was converted into Na mapping image by ImageJ version 1.51j8 (National Institutes of Health, USA). We checked the intensity of the Na signal in energy dispersive X-ray spectroscopy (EDX) images by quantitative analysis provided in the EMAX Evolution. Sodium mapping and signal intensity in these species were tested by more than three biological replicates.

### Acclimation test

To test the effect of acclimation on Na allocation, plants were grown for 2 weeks in hydroponic culture without NaCl, transferred to a culture with 50 mM NaCl for 3 days, and then transferred to that with 100 mM NaCl (with 5 kBq ^22^Na) for another 3 days. After the treatment, plants were collected and processed for autoradiography as described above.

### Acute salt stress

To test the effect of acute salt stress, plants grown in hydroponic culture without NaCl were transferred to that with 200 mM NaCl for 3 days. Plants were then visually evaluated for salt injury.

## Results

### Sodium allocation in *V. riukiuensis* and its relatives

**Fig. 1:**
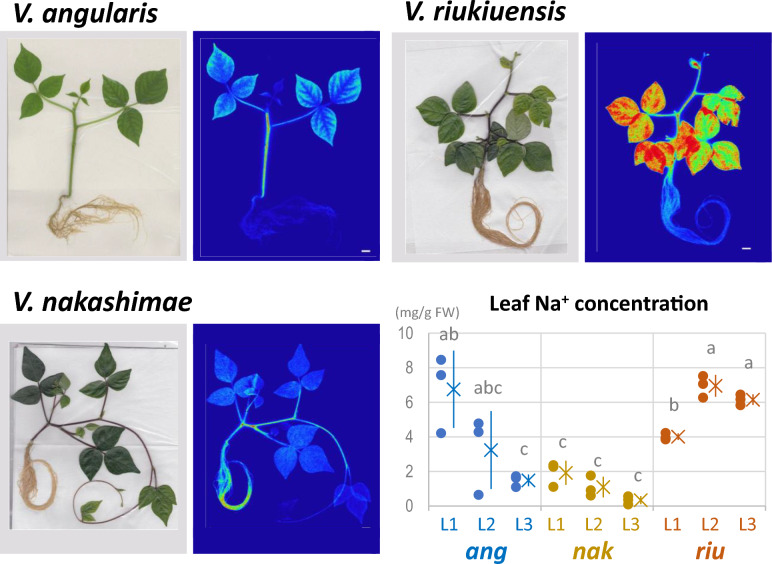
Na allocation in the plants. **a**–**c** Bright field photograph (left) and autoradiography (right) of the plants fed with ^22^Na. The luminance of ^22^Na distribution was standardized for each accession. Color change from blue to red indicates ^22^Na accumulation. White bars indicate 1 cm. **d** Na concentrations in each leaf. Circles, X and error bars indicate values of each replicate, means and standard deviation, respectively. L1, L2 and L3 indicate the 1st, 2nd and 3rd leaves, respectively. ang, nak and riu indicate *V. angularis*, *V. nakashimae* and *V. riukiuensis*, respectively. Means not sharing the same alphabet are significantly different (Tukey HSD *p* < 0.05)

To confirm that *V. riukiuensis* accumulates higher amount of Na in the leaves, we evaluated Na allocation in *V. angularis*, *V. nakashimae* and *V. riukiuensis* by autoradiograph and mass spectrometry. The results reproduced our previous observations (Noda et al. 2022; Yoshida et al. 2016), where Na allocation to the leaves were low in *V. nakashimae* but high in *V. riukiuensis* (Fig. 1). *V. angularis*, which is sensitive to salt stress, allocated more Na in the lower leaves than in the higher leaves.

### Electron microscope image of leaf cells

Because *V. riukiuensis* accumulated a higher amount of Na in the leaves, we considered that it isolated Na to the vacuoles. To test whether any specific features are observable in the vacuoles of *V. riukiuensis*, we obtained electron microscope images on the leaf cells of *V. angularis* and *V. riukiuensis*.

**Fig. 2:**
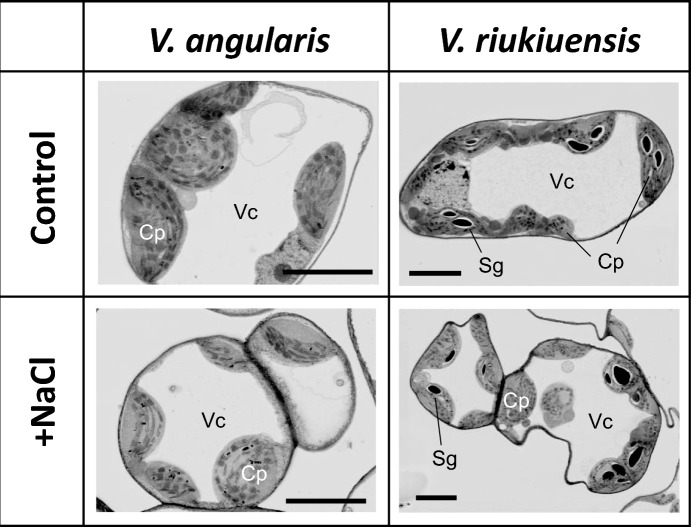
Electron microscopy images of leaf cells. Cp. Sg and Vc indicate chloroplast, vacuole and starch granules, respectively. Black bars indicate 5 µm

However, we could not find any specific features in vacuoles of *V. riukiuensis*. Regardless of salt stress, the vacuoles were fully developed and occupied most of the cell compartments in both the species (Fig. 2). The only difference we found between the two species was that *V. riukiuensis* contained well-developed starch granules in the chloroplasts whereas *V. angularis* did not (Fig. 2).

### Iodine–starch staining

**Fig. 3:**
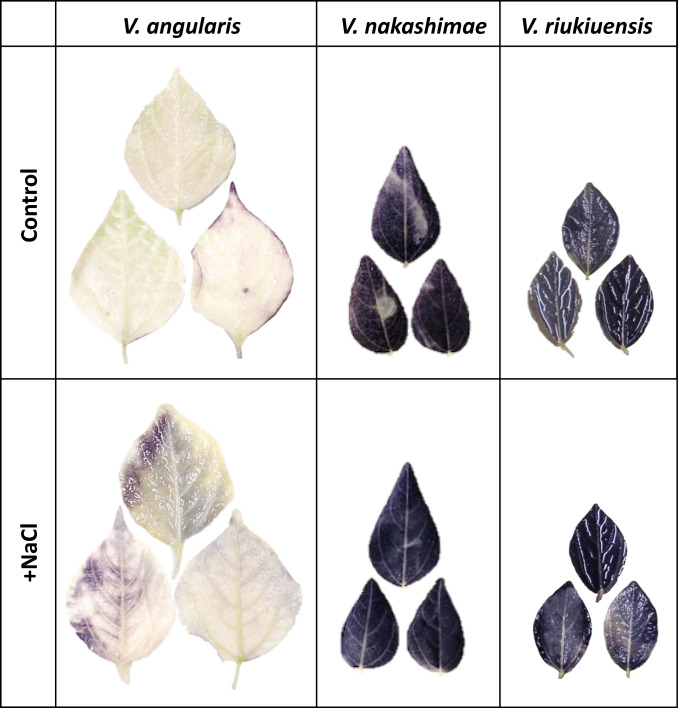
Iodine staining of the leaves. The 3^rd^ leaves of non-stressed or salt-stressed plants were stained with iodine

To confirm that *V. riukiuensis* accumulates starch granules in the leaf cells, we conducted iodine–starch staining on the leaves of *V. angularis, V. nakashimae* and *V. riukiuensis* (Fig. 3)*.* As a result, we observed strong staining in the leaves of *V. riukiuensis* but not in those of *V. angularis*, confirming high starch content in *V. riukiuensis* but low in *V. angularis.* However, we also observed strong staining in the leaves of *V. nakashimae*. Thus, it did not seem that the Na allocation to the leaves in *V. riukiuensis* was because of the higher content of starch. The starch granule experiment was conducted at the same time (2:00 p.m.), taking into account the difference in accumulation due to the day-night cycle.

### Suppressed Na allocation to shaded leaves of *V. riukiuensis*

**Fig. 4:**
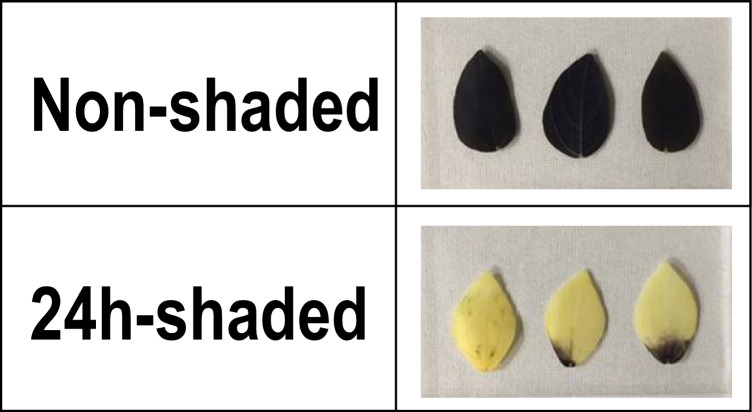
Effect of 24-h-shading on starch content in the leaves. Shaded and non-shaded leaflets were stained with iodine

However, we still suspected that starch granules in *V. riukiuensis* positively affected Na allocation to the leaves. To test the hypothesis, we shaded certain leaflets of *V. riukiuensis* plants for 24 h to have all the starch granules degraded (Fig. 4). We then fed the plants with ^22^Na and took an autoradiograph to visualize Na allocation. As a control experiment, we wrapped the leaflets of the same positions with cling film to minimize the effect of covering, which might reduce Na accumulation by decreasing transpiration.

**Fig. 5:**
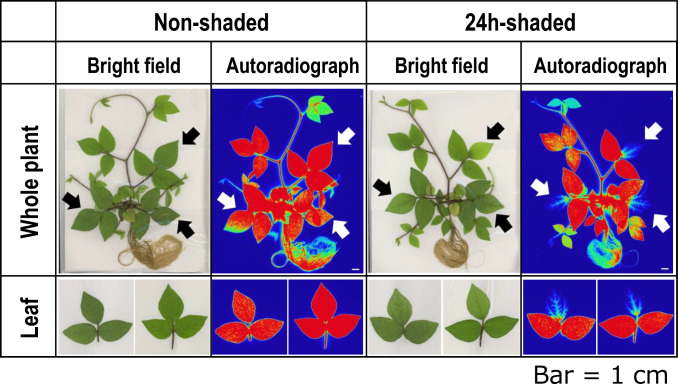
^22^Na accumulation in shaded and non-shaded leaves of *V. riukiuensis.* Black and white arrows indicate leaves that were wrapped with cling film (non-shaded) or foil (24 h-shaded). The luminance of each autoradiography was standardized. White bars indicate 1 cm

The results were remarkable. The autoradiography after ^22^Na treatment clearly showed that the shaded leaflets did not accumulate ^22^Na, while all other leaves (including “wrapped” leaflets in control) exhibited strong indication of ^22^Na allocation (Fig. 5).

### Scanning electron microscope-energy dispersive x-ray spectrometry (SEM–EDX)

**Fig. 6:**
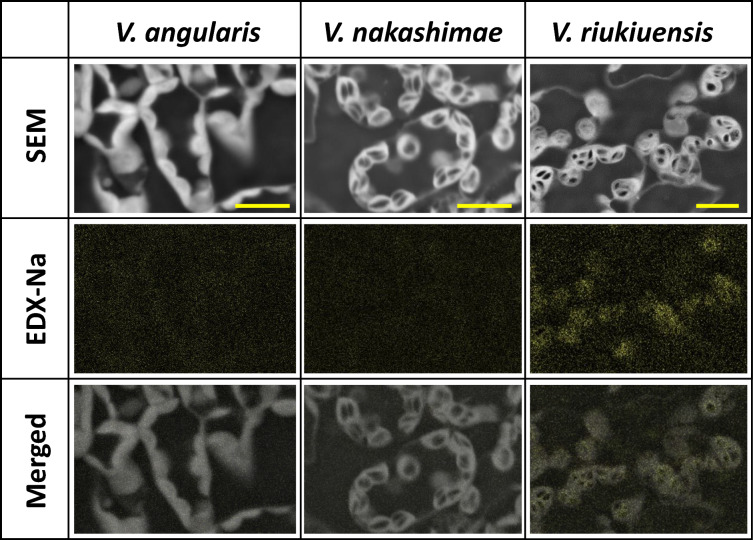
Electron microscopy (SEM), EDX (EDX-Na), and merged images of leaf cells*.* In the SEM images, the white structures in the cells are chloroplasts and the black objects inside them are starch granules. The yellow dots in EDX-Na images indicate the detected Na signals. Yellow bars indicate 1 µm

Although the result of the above experiment does not exclude the possibility that light signals trigger Na influx into the leaf cells, it motivated us to test whether the starch granules have the capability of binding Na in *V. riukiuensis*. To do so, we performed SEM–EDX to locate Na in leaf sections (Fig. 6). We should note that the process of preparing leaf sections (including fixation) washes free Na^+^ ions away from vacuoles, cytoplasm and apoplastic spaces. Thus, SEM–EDX can detect only Na bound to cellular components.

As expected from the results of iodine–starch staining experiments (Fig. 3), the SEM image showed that there were few starch granules in the leaf sections of *V. angularis* whereas they were abundant in those of *V. nakashimae* and *V. riukiuensis* (Fig. 6).

The following EDX analysis detected few Na in the sections of *V. angularis* and *V. nakashimae* but detected Na in the sections of *V. riukiuensis*, especially in chloroplasts (Fig. 6). Moreover, Na was not in the middle of starch granules but was enriched around them (Fig. 6). As such, in the leaves of *V. riukiuensis*, Na was isolated in chloroplasts, especially around starch granules.

### Effect of Na-binding on starch degradation

To elucidate if Na-bound starch granules were also degraded when shaded, we pre-treated the *V. riukiuensis* plants with 100 mM NaCl for 24 h, covered leaflets with aluminum foil for another 24 h and then conducted iodin-starch staining.

**Fig. 7:**
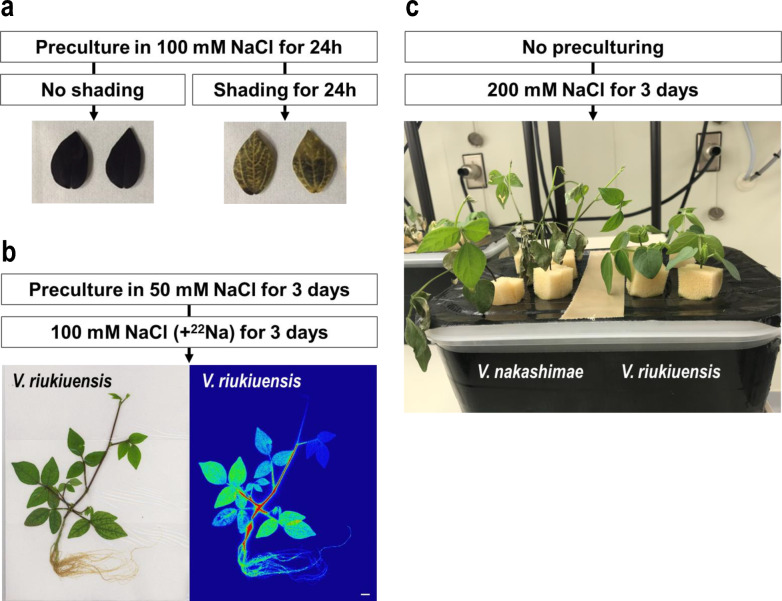
**a** Effect of Na-binding to starch degradation by shading.** b** Autoradiograph of *V. riukiuensis* plant fed with ^22^Na after acclimation.** c** *V. nakashimae* and *V. riukiuensis* plants under acute salt stress. Summary of experimental procedures are indicated in text boxes with arrows

As a result, the shaded leaflets still exhibited significant staining (Fig. 7a), which highly contrasted from those without pre-treatment (Figs. 4, 5).

### Na allocation in salt-acclimated plants of *V. riukiuensis*

As some plant species change patterns of Na allocation by salt acclimation (Noda et al. 2022), we also analyzed Na allocation in *V. riukiuensis* plants that were acclimated to salt stress. To do so, we pre-treated the plants with 50 mM NaCl without radioisotope for 3 days, fed 100 mM ^22^NaCl for another 3 days and then took autoradiographs.

As a result, the pattern of salt allocation of *V. riukiuensis* plants was similar to that of *V. nakashimae* (Figs. 1, 7b). Compared to the *V. riukiuensis* plant in Fig. 1, it allocated more Na to the root and the stems, and less to the leaves.

### Tolerance of *V. nakashimae* and *V. riukiuensis* to acute salt stress

Because *V. riukiuensis* reduced Na allocation to the leaves when acclimated, we were intrigued to test a hypothesis that the Na capture by starch granules buffer impact of Na uptake before the plant adjusts itself to the high-salt condition. Thus, we treated the plants of *V. nakashimae* and *V. riukiuensis* with 200 mM NaCl without any pre-treatment with lower concentration of NaCl.

The plants of both species exhibited symptoms of etiolation for a few hours because of the sudden decrease of osmotic pressure, but they all recovered within another few hours. Though the plants looked recovered from the osmostress, the leaves of *V. nakashimae* exhibited severe injury in 2 days, while those of *V. riukiuensis* did not exhibit any visual damage (Fig. 7c).

## Discussion

In this study, we demonstrated that *V.riukiuensis* accumulates starch granules that bind Na, which could be the second evidence of Na-binding starch granules in plants. The only report before this study was of common reed, which forms Na-binding starch granules in the shoot base in response to salt stress (Kanai et al. 2007). Kanai et al. have demonstrated co-localization of Na with starch granules and the isolated starch granules contained higher amounts of Na than other parts of the shoot base (Kanai et al. 2007). Although *V. riukiuensis* forms Na-binding starch granules in the leaves not in the shoot base (Figs. 1, 2, 3, 4, 5, 6), these facts suggest that the Na-trapping system by starch granules had independently evolved in multiple salt-tolerant species.

One may argue that our results in Fig. 5, where shading removed not only starch but Na allocation from the leaves, could be due to lack of light signals that may trigger Na influx into the leaf cells. Supporting such an argument, dark treatment suppressed the iron transport to the mesophyll cells in barley leaves (Bughio et al. 1997). We admit that our control experiment, where we covered the leaflets with cling films, excluded only the effect of transpiration on Na allocation to the leaves, but not the effect of light signals (Fig. 5). However, the previous report of Na-binding starch from common reed (Kanai et al. 2007) encouraged us to conduct SEM–EDX to directly test if the starch granules in *V. riukiuensis* are also able to bind Na. Fortunately, the SEM–EDX provided us the results that support our hypothesis (Fig. 6)*.*

Since we previously revealed that *V. riukiuensis* allocates relatively higher amount of Na to the leaves, we have considered it has an includer-type mechanism, which sequesters excess Na^+^ into vacuoles (Noda et al. 2022; Yoshida et al. 2016). However, recent studies argue that the vacuole is not an ideal organelle for Na^+^ sequestration, as the tonoplast is permeable to Na^+^ and thus allows back-leak to cytosol (Shabala et al. 2020). Thus, for halophytic species including *V. riukiuensis*, it would be better to have a mechanism other than, or in addition to, the one using the vacuole. Na-binding starch granules could be one such mechanism, though we were surprised to see *V. riukiuensis* allocating Na to chloroplasts (Fig. 6), as Na is toxic especially to photosynthetic activity (Matoh et al. 1986). However, given Na bound to starch was not washed away during the process of sample fixation and section preparation (Fig. 6), it could serve to sequester and detoxify Na from cytosols and even within chloroplasts.

However, one can imagine that *V. riukiuensis* is sacrificing carbon storage for Na sequestration. As observed in Fig. 7a, starch degradation by shading, which is completely done within 24 h in the control condition, is not completed under salt stress. This result suggests that Na-bound starch granules are more difficult for a plant to use as energy source. Thus, if a plant had depended on starch granules for Na sequestration throughout its whole life, it would cause increased energy cost. However, reasonably, *V. riukiuensis* stops allocating Na to the leaves when it is acclimated to salt stress. As shown in Fig. 7b, preculture with 50 mM NaCl for 3 days enhanced Na allocation to the root and the stems, which is more like *V. nakashimae* (Fig. 1).

If *V. riukiuensis* also has an ability to suppress Na allocation to the leaves, what is the merit of having Na-binding starch? We consider it could buffer Na toxicity at the initiation of salt stress. As also observed in *V. nakashimae* in our previous study (Noda et al. 2022), acclimation enhanced Na evacuation in the root and the stem (Fig. 7b). Thus, before acclimation is complete, Na evacuation by the root and the stem is not strong enough to suppress Na allocation to the leaves. However, Na flown into leaf cells can be trapped and detoxified with Na-binding starch granules. The tolerance of *V. riukiuensis* in acute salt stress might reflect such buffering effect of Na-binding starch (Fig. 7c).

This result also indicates that Na-binding activity is characteristic to the starch granules in *V. riukiuensis* and not to those of *V. nakashimae.* It was first complicating that *V. nakashimae* also accumulates lots of starch granules in the chloroplasts but does not allocate Na to the leaves. It is still possible that *V. nakashimae* has an exceptional ability to completely exclude Na^+^ out of the leaves or that its chloroplasts simply do not incorporate lots of Na, but the sensitivity to the acute stress (Fig. 7c) does not support these ideas. Thus, the starch granules in *V. nakashimae* probably lack Na-binding ability.

Although pure amylose chains do not have any ability to bind cations including Na^+^, modification of hydroxyl groups such as phosphorylation turns them into cation exchangers (Matsumoto et al. 1998). Given amylose chains are extended from the surface of starch granules (Goren et al. 2018), *V. riukiuensis* and common reed may have higher enzymatic activity in modifying those chains. In addition, other studies also report that α-glucan-like molecules in common reeds bind cadmium (Cd) ions and reduce Cd stress (Higuchi et al. 2013, 2015). As such, some plant species have acquired abilities to produce and accumulate ion exchangers to attenuate sodium and other metal ion stresses.

Lastly, we mention the environmental difference between the habitats of *V. riukiuensis* and *V. nakashimae*, which may explain the importance of Na-binding starch in *V. riukiuensis.* Though both species live in coasts of remote islands, those of *V. riukiuensis* are located in the tropics (Iseki et al. 2016) and are frequently hit by typhoons. Thus, compared to *V. nakashimae*, whose habitat is located in temperate areas (Iseki et al. 2016), *V. riukiuensis* is often exposed to acute salt stress by high tide and sea spray. Such an environment might have selected the higher tolerance of *V. riukiuensis,* to which Na-binding starch could contribute.

To conclude, we have demonstrated common reed is not the only species to have evolved Na-binding starch granules. As it is effective in multiple plant taxa, we are intrigued to apply this system for developing salt-tolerant crops. By combining other mechanisms of salt tolerance, it would bring synergistic effects on salt tolerance.

## Data Availability

Data are available on request from authors.
